# Signal intensity loss of the intervertebral discs in the cervical spine of young patients on fluid sensitive sequences

**DOI:** 10.1007/s00256-015-2301-7

**Published:** 2015-12-04

**Authors:** F. de Bruin, S. ter Horst, R. van den Berg, M. de Hooge, F. van Gaalen, K. M. Fagerli, R. Landewé, M. van Oosterhout, J. L. Bloem, D. van der Heijde, M. Reijnierse

**Affiliations:** Department of Radiology, C2-S, Leiden University Medical Center, Albinusdreef 2, PO box 9600, 2300 RC Leiden, The Netherlands; Department of Rheumatology, Diakonhjemmet Hospital, Oslo, Norway; Department of Rheumatology, Amsterdam Medical Center, Amsterdam, The Netherlands; Department of Rheumatology, Groene Hartziekenhuis, Gouda, The Netherlands; Department of Rheumatology, Leiden University Medical Center, Leiden, The Netherlands

**Keywords:** Signal intensity of intervertebral discs, Cervical spine, Fluid sensitive MR

## Abstract

**Objective:**

To evaluate the signal intensity (SI) of the intervertebral discs of the cervical spine on magnetic resonance (MR) fluid sensitive sequences, and correlate this to secondary signs of degeneration on MR and radiographs as well as to age.

**Material and methods:**

A total of 265 patients aged ≥16 with back pain (≥3-months, <2-year, onset <45-years) from the SPondyloArthritis Caught Early (SPACE) cohort were included. Sagittal 1.5 T MR images and lateral radiographs of the cervical spine were independently evaluated by two readers for: SI of the intervertebral discs using a grading system based of Pfirrmann (grade 1 normal/bright SI; 2 inhomogeneous/bright SI; 3 inhomogeneous/mildly decreased SI; 4 inhomogeneous/markedly decreased SI; 5 signal void), disc herniation and Modic changes (MRI) and disc space narrowing, osteophytes and sclerosis (radiograph). Readers were blinded for clinical information. Descriptive statistics were used for characteristics and prevalence of findings, and regression analysis was used for age and grades.

**Results:**

Of 265 patients (36 % male, mean age 30), 221 (83 %) patients had 1 to 6 discs (median 4) with decreased SI. Of 1,590 discs, 737 (46 %) were grade 1; 711 (45 %) grade 2; 133 (8 %) grade 3; 8 (1 %) grade 4 and 1 (0 %) grade 5. Secondary signs of degeneration were rare and seen predominantly in C5–C7 and appear to be related to signal loss grade 3 and 4.

**Conclusion:**

Low signal intensity of intervertebral discs in absence of secondary degenerative signs in the cervical spine on fluid sensitive MR images might be pre-existing and part of the natural course.

## Introduction

**Fig. 1:**
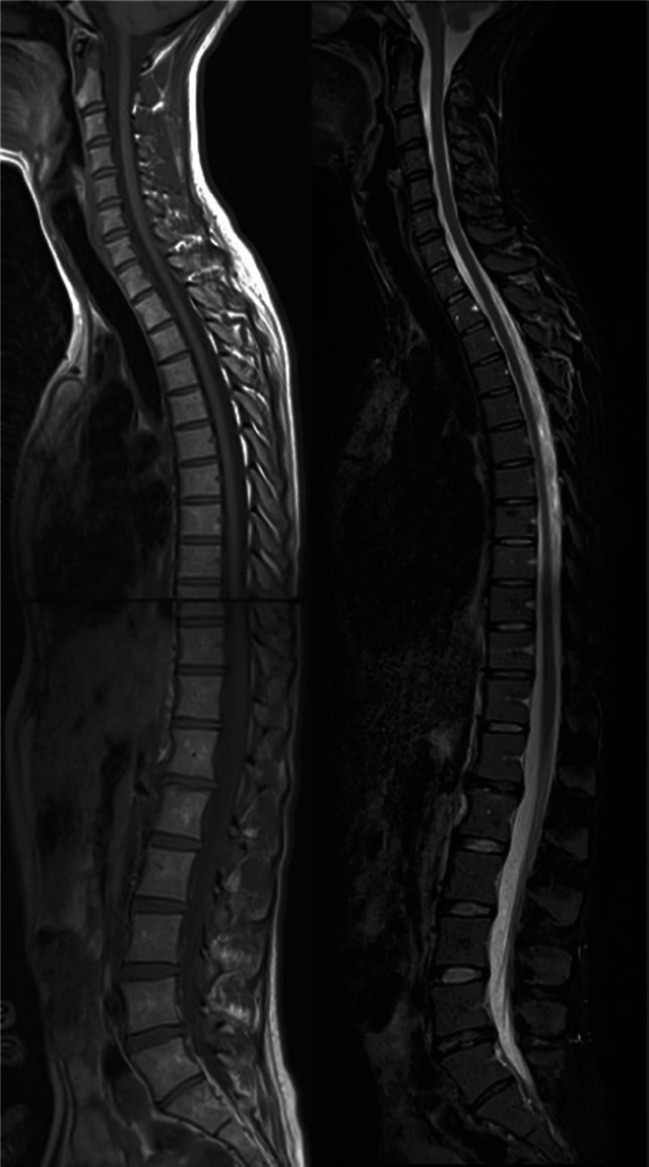
Sagittal T1 and STIR image of the whole spine. On the STIR, grade 2–3 intervertebral discs at levels C2–C7 are observed, in the absence of secondary degenerative changes. In addition, at levels L1–L2 and L5–S1 intervertebral discs with high grades are appreciated in the presence of height loss and disc bulging. The thoracic spine is normal

Degenerative changes of the spine, especially of the intervertebral discs, are a common finding in normal individuals without clinical symptoms. One study reports in 558 patients aged 20–22 year olds a prevalence of 47 % of subjects with disc degeneration in at least one lumbar disc [1]. When evaluating the SPondyloArthritis Caught Early (SPACE) cohort (a large cohort of young patients, age range 16–46) with chronic back pain of short duration) [2], we came across a number of patients with signal loss in intervertebral discs of the cervical spine during assessment of MRI of the spine (Fig. 1). Several studies have reported about the prevalence of degenerative changes in the cervical spine [3–6], and used various grading systems—e.g., one study graded discs as bright (grade 0), dark and/or speckled (grade 1) and almost black (grade 2). However, to our knowledge, there is no standard of reference for the normal signal intensity of the intervertebral discs in the cervical spine. In general, the Pfirrmann grading is used for grading signal changes and disc height on T2-weighted images of intervertebral discs in the lumbar spine; therefore, the aim of this study is to evaluate the signal intensity and height of the intervertebral discs in the cervical spine using the description of the Pfirrmann grading system in a young cohort of patients with chronic back pain of short duration. In addition, we correlate these findings to secondary degenerative changes on MRI and radiographs as well as to age.

## Patients and methods

The SPACE cohort study is an ongoing cohort, performed with the approval of the institutional review board and all participants provided written informed consent. The SPACE cohort is a multicentre study, set up to investigate the early stages of axial spondyloarthritis (axSpA) [2]. Inclusion criteria are chronic back pain of unknown origin longer than 3 months but no longer than 2 years and age between 16 and 45 years at onset of back pain. At baseline, patients underwent clinical, laboratory and imaging assessment and patients were asked in what part of the spine they experienced pain; in the buttock, the lumbar spine, the thoracic spine, a combination of the three or in the total spine. Patients with pain in the total spine potentially had symptoms in the cervical spine and were excluded for the current study. Patients were classified according to Assessment of SpondyloArthritis international Society (ASAS) axial SpA criteria into no axSpA patients (no SpA features at all), possible axSpA patients (not fulfilling ASAS axSpA criteria but with one or more SpA features) and axSpA patients (fulfilling ASAS axSpA criteria) [7]. Sagittal T1-weighted turbo spin echo (Repetition time/Echo time; T1TSE; TR 550/TE 10) and short tau inversion recovery (STIR; TR 2500/TE 60) sequences of the whole spine were acquired on a 1.5 T MRI (Philips Medical Systems, Best, The Netherlands). The slice thickness was 4 mm. Lateral cervical spine radiographs were performed with patients erect and the cervical spine in neutral position. Data obtained at the baseline visit of patients included between January 2009 and December 2012 were used in the current analysis [2].

### Image evaluation

For this analysis, levels C2–Th1 were evaluated (6 disc levels per patient, 1,590 discs in total). All images were independently scored by two trained readers (StH, a musculoskeletal radiologist and FdB, a trained reader), blinded for clinical information as well as the other imaging modality. In case of disagreement between the readers, a third reader (MR, an experienced musculoskeletal radiologist) acted as adjudicator. In case of a difference in 1 grade, the lower score was used. In case the two initial readers disagreed on more than 1 grade, the adjudicator’s score was used.

On MR the following parameters were scored: signal intensity of the intervertebral discs (C2–Th1) on STIR images on a 5 point scale (grade 1 normal bright signal intensity; grade 2 inhomogeneous bright signal intensity; grade 3 inhomogeneous mildly decreased signal intensity; grade 4 inhomogeneous markedly decreased signal intensity; grade 5 signal void; Table 1) [8]; disc herniation as either protrusion or extrusion [9]; the presence/absence of Modic changes—type I: bone marrow edema (BME), defined as high signal of both adjacent vertebral endplates on STIR images; type II: fatty changes defined as high signal of the vertebral endplates on T1-weighted images; type III: sclerosis defined as low signal of the vertebral endplates on T1 and STIR images [10]. On radiographs we scored for: the presence or absence of disc space narrowing; defined as narrowing of the disc space in comparison to two healthy adjacent levels; the presence or absence of osteophytes: defined as bone hypertrophy arising from the vertebral body close to the vertebral endplate, and with a maximum of 45° between the bony spur and the endplate (to differentiate from syndesmophytes) and the presence or absence of sclerosis defined as increased bone density adjacent to the vertebral endplates.

**Table 1:**
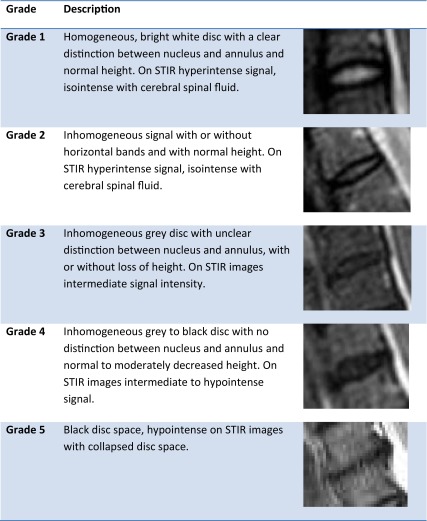
Grading of signal intensity of the intervertebral disc of the cervical spine on STIR images (modified from the Pfirrmann classification)

Statistics: Statistical analysis was performed based on adjudicated scores. Continuous data is reported using mean values and the standard deviation (SD), categorical data is reported as range and median and frequencies are reported as percentages. Regression analysis was used to test for association between age and grade. Cohen’s kappa was used to test interobserver agreement (weighted kappa for grading).

## Results

In total, 265 patients (36 % male, mean age 30.0 ± 8) with 1,590 intervertebral discs were evaluated. Of these, 107 patients (40.4 %) had axSpA, 24 (9.1 %) patients had no axSpA and 134 (50.6 %) had limited features of axSpA not fulfilling the ASAS axSpA criteria. Of 265 patients, 22 (8.3 %) had pain only in the buttocks, 102 (38.5 %) in the lumbar spine and 25 (9.4 %) in the thoracic spine. One hundred and 16 (43.8 %) patients had pain at multiple sites of buttock, lumbar and thoracic spine.

**Fig. 2:**
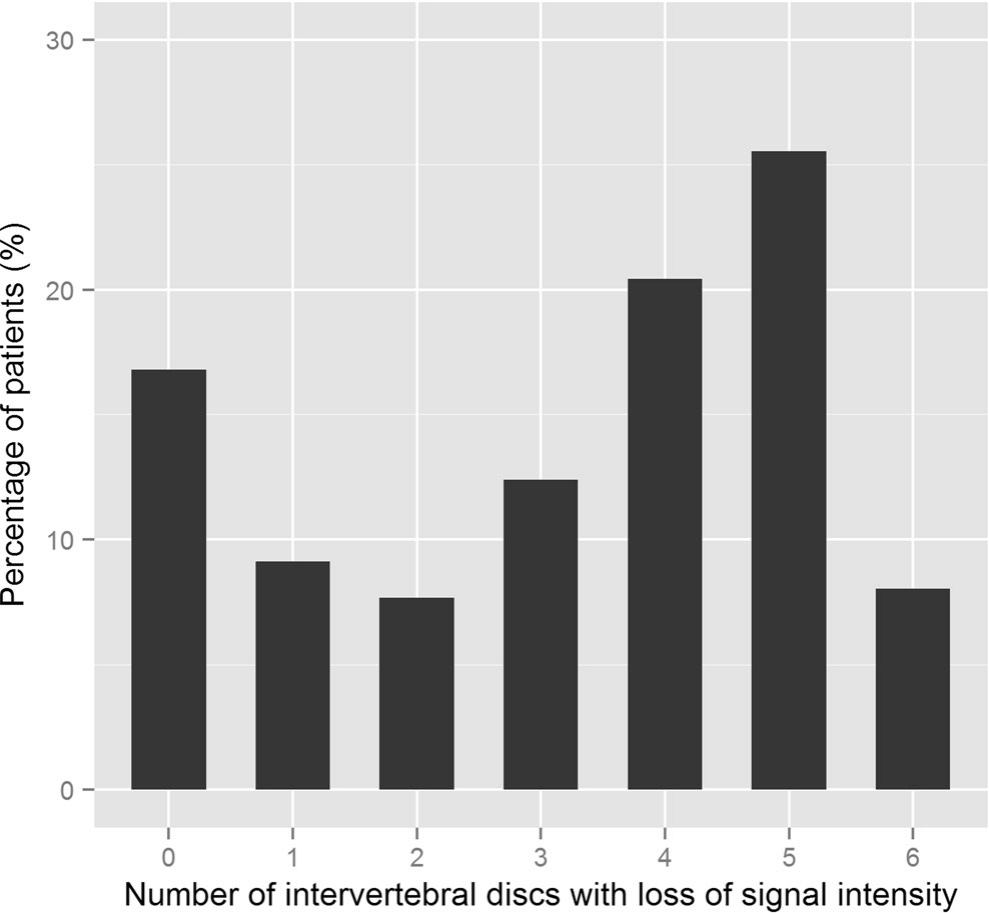
Percentage of intervertebral discs of the cervical spine with loss of signal intensity (Grade class > 1) per patient

Interobserver agreement was substantial for grading (*κ* = 0.73), Modic changes (*κ *= 0.80), disc herniation (*κ* = 0.68), disc height on radiographs (*κ* = 0.69) and osteophytes (*κ* = 0.74) and almost perfect for sclerosis (0.92). Adjudication was performed in 16 patients for grading. Of 265 patients, 44 patients (16.6 %) had no intervertebral discs with loss of signal intensity. Of the remaining 221 patients, 23 patients (8.7 %) had 1 disc with loss of signal intensity, 20 (7.5 %) patients had 2 and 178 patients (67.2 %) had 3 or more discs with loss of signal intensity. In the whole population, the range of affected discs was 0–6 with a median of 4 (Fig. 2).

**Fig. 3:**
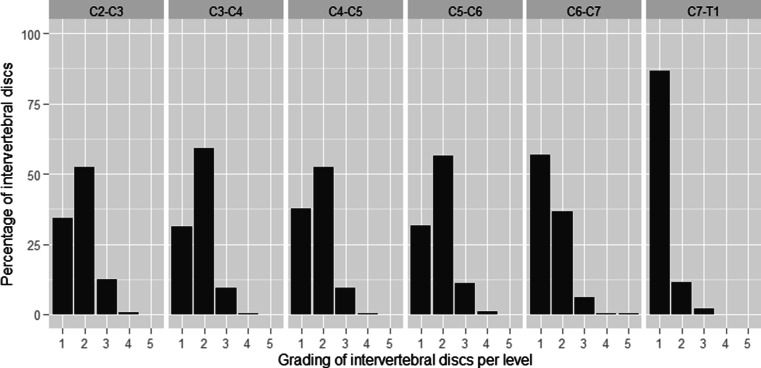
Distribution of grades per intervertebral disc of the cervical spine

For levels C2–C3 through C5–C6, a grade 2 was scored in 138/265 (52.1 %), 155/265 (58.5 %), 138/265 (52.1 %) and 148/265 (55.8 %) patients, respectively (Fig. 3) and grade 3 was scored in 33/265 (12.5 %), 25/265 (9.4 %), 25/265 (9.4 %) and 29/265 (10.9 %) patients, respectively. In total, 128 patients (48.3 %) had signal loss in all first four discs. At level C6–C7, 96/265 (36.2 %) had grade 2 and 16/265 (6.0 %) had grade 3, respectively. For level C7–T1, grade 1 was observed in 228/265 patients (86.0 %), grade 2 in 30/265 patients (11.3 %) and grade 3 in 5/265 (1.9 %) patients, respectively. Eight discs with grade 4 were observed in 4 patients (1.5 %) and grade 5 was scored once in one patient.

Of all 1,590 intervertebral discs, 737 (46.3 %) were graded as grade 1, 711 (44.7 %) were scored as grade 2, 133 (8.4 %) as grade 3, 8 (0.5 %) as grade 4 and 1 (0 %) as grade 5. Secondary degenerative signs on MRI and radiographs were seen throughout the cervical spine, with the highest prevalence in C5–C6 and C6–C7. On MRI, Modic I was seen in 1/1,590 disc (0.1 %), Modic II in 3/1,590 discs (0.2 %) and disc herniation in 46/1,590 discs (2.9 %). On radiographs, loss of disc height was seen in 58/1,590 discs (3.6 %), osteophytes in 20/1,590 discs (1.3 %) and sclerosis in 5/1,590 (0.3 %) discs. Modic type III was not observed. Secondary signs were predominantly associated with discs graded 3 and 4 (Table 2). For all levels except C7–T1, age was statistically significant associated with intervertebral disc grading. *β* ranged from 0.02 (C3–C4) to 0.04 (C6–C7), *P* < 0.001.

**Table 2:** Intervertebral disc grading correlated to signs of degeneration on MR and radiographs of the cervical spine

Sign of degeneration	Intervertebral disc grade *n* = 1590 (%)				
	Grade 1	Grade 2	Grade 3	Grade 4	Grade 5
	*n* = 737 (47)	*n* = 711 (45)	*n* = 133 (8)	*n* = 8 (1)	*n* = 1 (0)
MRI					
Modic type I	0	1 (0)	0	0	0
Modic type II	0	1 (0)	2 (2)	0	0
Disc bulging	2 (0)	36 (5)	7 (5)	2 (25)	0
Radiograph					
Loss of disc height	12 (2)	31 (4)	13 (10)	1 (13)	1 (100)
Osteophytes	0	12 (2)	7 (5)	1 (13)	0
Sclerosis	0	4 (1)	1 (1)	0	0

Percentages of degenerative findings in between brackets, as a proportion of the specific intervertebral disc grading

## Discussion

In this study, we demonstrated that signal loss of intervertebral discs in the cervical spine is common in young (mean age 30.0 years) subjects. In less than 20 % of 265 patients included in this study, the cervical spine was without loss of signal (Fig 2). A marked difference between the upper levels (C2–C3 to C5–C6) relative to the lower levels C6–C7 and C7–T1 is seen (Fig 3). In the upper levels, grade 2 was more often found than grade 1. Levels C6–C7 and especially C7–T1 showed more discs with normal signal intensity than reduced signal.

MR signal intensity of intervertebral discs in the cervical spine has been debated. Boden et al. [11] reported in 1990 on MR scans in 63 asymptomatic subjects (mean age 40, range 20–73) that 19 % of scans showed degenerative changes (disc herniation, disc space narrowing, disc degeneration, osteophytes and/or spinal cord compression) in the cervical spine and that these should be always correlated to clinical symptoms before therapeutic decisions are made. In another study reported in 1998 [6], the discs of 497 asymptomatic subjects were graded bright (grade 0), dark and/or speckled (grade 1) and almost black (grade 2). Of these 497 patients, 170 patients matched our population (age between 20 and 39 years). In these patients, up to 30 % of discs were scored as grade 1 and 10 % as grade 2. We decided to use a modified version of the well-known Pfirrmann grading system because a 5-points scale may better distinguish between different stages of degeneration compared to the 3-point scale used by Matsumoto. Advances in imaging techniques and a more detailed grading system might explain the difference between these studies and the current study. Hayashi found in the cervical spine of 437 symptomatic patients (mean age 49.8 ± 10) that disc degeneration was comparable in the levels C3 through C6 and that secondary signs (Modic changes) were found in the levels C5-C7 with highest prevalence, as was the case in our study [12]. Some authors reported on changes in cervical intervertebral disc signal intensity as a sign of traumatic damage and cause of pain in whiplash patients [13, 14]. In 2014, Ulbrich showed in 50 whiplash patients (50 % male, mean age 35 years, range 19–61) and 50 age- and gender-matched control subjects, that signal intensity was not affected after a follow-up period of 6 months, and showed a significant effect of age and disc level on signal intensity. C6–C7 was reported to increase most in grade with increasing age, as was the case in our study.

The intervertebral disc connects the individual vertebral bodies into the spine and plays an important role in the biomechanics of the spine [15]. In contrast to the thoracic and lumbar spine, the cervical spine is a highly mobile part of the axial skeleton, and flexion-extension and rotation are the main movements. Cervical discs are structurally different from lumbar discs; no concentric annulus fibrosus is present (the posterior part is not developed) and the nucleus pulposus is relatively small, partially explaining the generalized lower signal intensity in the cervical discs presented in the current study [16, 17]. With aging and degeneration, the water content of the nucleus pulposes is further reduced and fibrous tissue is formed [3, 17–19], which causes the signal intensity of the intervertebral discs to change from high to low on MR fluid sensitive images. Although a study by Roberts et al. in 1997 suggests an increase of the intervertebral disc height due to microfracturing and remodelling of the vertebral endplate [20], more evidence supports the opposite: it has been demonstrated that eventually, when enough water content disappears from the nucleus pulposus, the disc looses height and becomes stiffer [15]. Simultaneously (but before the discus becomes too stiff) repetitive mechanical loading can lead to disc prolapse and end plate damage.

However, there is a remarkable difference in the distribution of signal loss of the intervertebral disc in the cervical spine compared to the lumbar spine. In the lumbar spine, disc degeneration is concentrated in the lower part of the lumbar spine (L4–L5 and L5–S1 discs) and with much higher prevalence of secondary signs [21–24], while results of the current study and other studies show that mild loss of signal intensity of the discs in the cervical spine is more evenly distributed, with few secondary changes [3, 11, 12].

We hypothesize that this is due to high mobility (distributing the shear forces over multiple discs) and low weight bearing of the cervical spine, in contrast to the lumbar spine where there is low mobility and high gravitational forces [15, 25]. In our study, a tendency towards secondary degenerative signs in the presence of a higher grade of intervertebral disc signal intensity on MR is observed; however, numbers in the groups with high grades were too small for statistical analysis.

Although age was statistically significantly associated with higher intervertebral disc grading, the increase with age within our young population was small (*β* did not exceed 0.04, which means an increase of one grade in 25 years assuming linearity). The higher intervertebral disc grading might increase more substantially in an older population, however, no data are available to confirm this.

Our study had a number of limitations. We used a subjective grading instead of an objective measurement (e.g., quantitative measurement via region-of-interest signal measurement). On the other hand, our visual grading system can be easily used in daily clinical practice. We based our subjective grading on the Pfirrmann system that was used for the lumbar spine using T2-weighted images. We used another fluid sensitive sequence (STIR) with a lower SNR than T2-weighted images with frequency selective fat suppression, but with similar sensitivity to fluid. In addition, we graded the cervical spine instead of the lumbar spine. We therefore described our grading system as based on the Pfirrmann grading system. Also the subjective scoring may have a lower reproducibility than a quantitative method. Lastly, we have not used an asymptomatic population with regard to the spine; 40 % of the patients in the current study fulfilled the ASAS axSpA criteria and 51 % had limited signs of axSpA without fulfilling the criteria; however, pain at the cervical level was an exclusion criterion.

## Conclusions

The number of intervertebral discs in the cervical spine with loss of signal on STIR in the absence of secondary signs of degeneration was high in our cohort of young patients. Therefore, radiologists and physicians requesting MR studies of the cervical spine should be aware of the fact that low signal intensity of intervertebral discs, or dehydration, in the cervical spine might be pre-existing and part of the natural course, even in young patients.
